# The nature of magnitude integration: Contextual interference versus active magnitude binding

**DOI:** 10.1167/jov.22.11.11

**Published:** 2022-10-19

**Authors:** Irene Togoli, Domenica Bueti, Michele Fornaciai

**Affiliations:** 1 International School for Advanced Studies (SISSA), Trieste, Italy

**Keywords:** magnitude perception, magnitude integration, time perception, numerosity perception, magnitude binding

## Abstract

Magnitude dimensions such as duration and numerosity have been shown to systematically interact, biasing each other in a congruent fashion: the more numerous a set of items is, the longer it is perceived to last in time. This integration between dimensions plays an important role in defining how we perceive magnitude. So far, however, the nature of magnitude integration remains unclear. Is magnitude integration a contextual interference, occurring whenever different types of information are concurrently available in the visual field, or does it involve an active “binding” of the different dimensions of the same object? To address these possibilities, we measured the integration bias induced by numerosity on perceived duration, in two cases: with duration and numerosity conveyed by distinct stimuli, or by the same stimulus. We show that a congruent integration effect can be observed only when the two magnitudes belong to the same stimulus. Instead, when the two magnitudes are conveyed by distinct stimuli, we observed an opposite effect. These findings demonstrate for the first time that a congruent integration occurs only between the dimensions of the same stimulus, suggesting the involvement of an active mechanism integrating the different dimensions of the same object in a unified percept.

## Introduction

Magnitude information encompasses a set of perceptual dimensions that are essential to understand the external environment. The processing of magnitude information indeed allows the brain to define how many objects are around us, how big they are, and how long and frequent the events occurring in the surrounding environment are. Interestingly, converging evidence shows that different magnitude dimensions are not perceived independently from each other, but are integrated in a way that the perception of one dimension depends on the other dimensions. For example, the bigger a stimulus is in size, the longer it is perceived to last in time (Cai & Connell, 2015; Rammsayer & Verner, 2014; Xuan, Zhang, He, & Chen, 2007). Similarly, a numerous set of items is perceived as lasting longer in time compared to a less numerous set, and a longer stimulus can be perceived as more numerous (Cappelletti, Freeman, & Cipolotti, 2009; Javadi & Aichelburg, 2012; Togoli, Fornaciai, & Bueti, 2021; Xuan et al., 2007). Moreover, different magnitude dimensions are similarly sensitive to distorting effects such as motion adaptation, again suggesting a link between the representation of different dimensions (Fornaciai, Arrighi, & Burr, 2016; Fornaciai, Togoli, & Arrighi, 2018; Turi & Burr, 2012).

These mutual biases across different dimensions are referred to as “magnitude integration” effects (Togoli et al., 2021) and have been proposed to reflect the operation of a generalized brain magnitude system, encoding and processing different dimensions with the same neural code (Walsh, 2003). According to “A theory of Magnitude” (ATOM; Walsh, 2003), the brain is indeed endowed with a generalized system for the processing of different magnitude dimensions, integrating them to more efficiently drive behavior (see for instance Skagerlund, Karlsson, & Träff, 2016 for evidence of overlapping brain areas processing different magnitudes). However, a generalized system is not a necessary condition for magnitude integration to occur, and magnitude processing could similarly be implemented by partially overlapping but parallel channels that do not converge onto a central mechanism. This view is for instance supported by neuroimaging results showing the lack of a common neural code for the representation of different dimensions (Borghesani, de Hevia, Viarouge, Pinheiro-Chagas, Eger, & Piazza, 2019), and the existence of overlapping but different cortical maps representing different magnitudes (Harvey, Klein, Petridou, & Dumoulin, 2013; Harvey, Fracasso, Petridou, & Dumoulin, 2015). This latter result, though, does not rule out the implementation of a common magnitude code. Besides the existence of a generalized processing mechanism or multiple overlapping magnitude channels, how magnitude integration itself works is largely unknown. For instance, it is unclear what the nature of magnitude integration is and what the conditions leading to a congruent integration between different dimensions are.

In the present study, we test a new hypothesis concerning the phenomenon of magnitude integration, based on the existence of a magnitude “binding” process integrating the different dimensions of a stimulus in a unified percept. According to this hypothesis, the interaction between different dimensions would occur in the process of generating a unified representation of a given stimulus, binding together its spatial, temporal, and numerical properties in the same way color and shape are bound to a unified object representation (Duncan, 1984; Gray, König, Engel, & Singer, 1989; Parto Dezfouli, Schwedhelm, Wibral, Treue, Daliri, & Esghaei, 2021; Treisman & Gelade, 1980). Magnitude integration might indeed be subject to the same “binding problem” that affects object perception more in general (i.e., the need to correctly identify to which stimulus a given dimension belongs to, in order to bind the dimensions belonging to the same object in a unified percept). In line with this, we thus predict that a congruent magnitude integration should occur exclusively when two dimensions belong to the very same stimulus. Alternatively, magnitude integration could simply be a contextual interference, occurring whenever two different types of information (e.g., numerosity and duration) are concurrently processed (Borghesani et al., 2019; Bueti & Walsh, 2009; Walsh, 2003) or stored in memory (Cai, Wang, Shen, & Speekenbrink, 2018). If this is the case, then the bias should similarly emerge irrespective of whether the different dimensions belong to the same or to distinct visual objects. We tested this new hypothesis in two independent experiments. In Exp. 1, we measured the effect of numerosity on perceived duration in two distinct cases: with duration and numerosity conveyed by two distinct stimuli (i.e., a texture marking the onset and offset of an interval, flashed on top of a dot array with a given numerosity) or by the very same stimulus (i.e., the dots in the dot array blinking to mark the onset and offset of the interval). In Exp. 2 we further investigated the properties of the magnitude integration effect in these two cases, to better understand the conditions leading to a congruent integration or to an opposite effect, and to exclude the possibility of attention playing a role in the observed effects. Namely, we first assessed the spatial selectivity of the effect when duration and numerosity are conveyed by distinct stimuli, by displacing the array of dots away from the texture marking the interval (Exp. 2a). Moreover, in the case of magnitudes conveyed by the same stimulus, we assessed whether the entire array of dots needs to convey the duration in order to observe an integration effect (i.e., all the dots blinking to mark the onset and offset of the interval), or only a subset of the dots conveying duration is sufficient to induce an effect, as long as the set is a part of the array (Exp. 2b).

## Methods

### Participants

A total of 46 subjects participated in the study. In Exp. 1, two groups of 15 participants each performed the two experimental conditions (Exp. 1a and Exp. 1b). In Exp. 2, 20 subjects took part in both experimental conditions (Exp. 2a and Exp. 2b). The group was composed of 13 males and 33 females with age ranging between 19 and 36 years (*M* = 24.76, *SD* = 3.57). The inclusion criteria for the study required participants to have normal or corrected-to-normal vision, and the absence of neurological, psychiatric and developmental disorder. The participants were tested separately, signed an informed consent form before participating in the study, and received a monetary compensation of 8€/hour. One participant was excluded from data analysis in Exp. 2 due to poor performance (see *Data analysis*). Note that the total number of subjects does not match the summed sample sizes of the different experiments because a few subjects (four) participated in both experimental conditions of Exp. 1. All the experimental procedures were approved by the ethics committee of the International School for Advanced Studies and were in line with the Declaration of Helsinki.

In Exp. 1, the sample size was based on a previous study from our group, where we tested a total of 15 participants (Togoli et al., 2021). In Exp. 2, because we used a smaller numerosity range, we instead performed a power analysis to determine the optimal sample size. Namely, we used the data from the same previous study (Togoli et al., 2021) to compute the expected effect size (Cohen's *d*) of the magnitude integration bias (effect of numerosity on perceived duration) considering only the smaller levels of numerosity (30 vs. 46 dots) tested in that experiment. The estimated effect size was 0.94. Considering a two-tailed distribution and a power or 95%, the estimated minimum sample size was 17 subjects, which we rounded up to 20 subjects to be conservative. The study was not preregistered.

### Apparatus and stimuli

Stimuli were created using the Psychophysics Toolbox (Kleiner, Brainard, & Pelli, 2007; Pelli, 1997) (version 3) for Matlab (r2020b, The Mathworks, Inc.) and displayed on a 1920 × 1080 LCD monitor (running at 120 Hz), encompassing a visual angle of 47° × 30° from a viewing distance of 57 cm. At this viewing distance, 1° of visual angle encompassed approximately 43 pixels.

**Figure 1.**
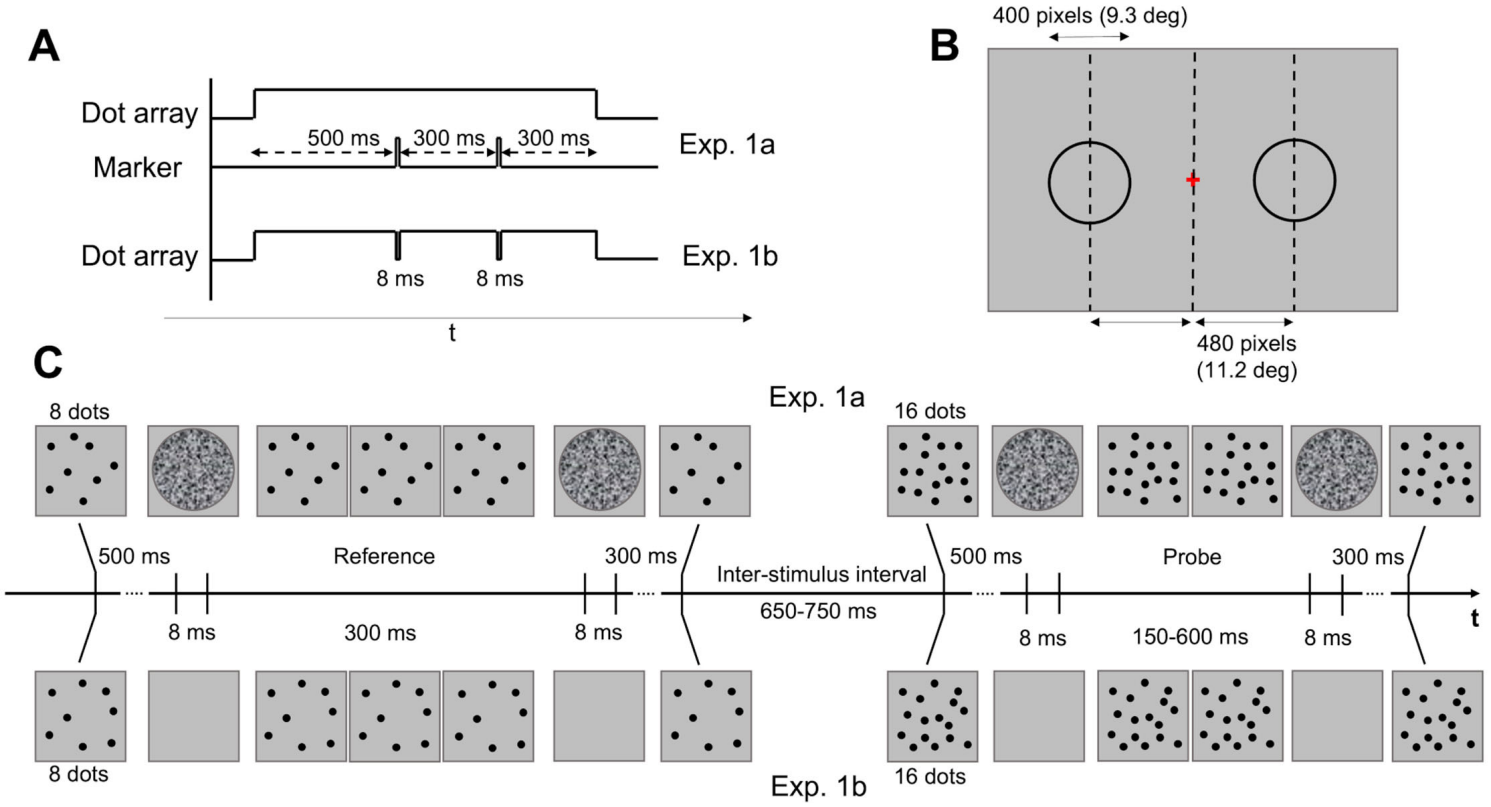
Stimuli and experimental procedure of Experiment 1. (A) Temporal dynamics of the reference stimuli in Exp. 1a (upper part) and Exp. 1b (lower part). The dot-array stimulus appeared on the screen 500 ms before the onset of the first marker and disappeared 300 ms after the second marker. (B) Position of the stimuli on the screen. Reference and probe stimuli were presented sequentially on the screen in two possible positions, either on the left or on the right of a central fixation point (distance = 11.2°). The order and position of the stimuli was randomized across trials. (C) Stimulation procedure (example of a single trial). The upper row shows the procedure of Exp. 1a, whereas the bottom row shows the procedure of Exp. 1b. Note that these examples show the case where the reference numerosity was eight dots, but in both Exp. 1a and 1b the reference numerosity could be either 8 or 32 dots. Stimuli are not depicted in scale. *Dots* in the actual experiment were black and white.

In all experiments, the dot-array stimuli were composed by a set of pseudo-randomly positioned black and white dots (in equal proportion; 100% contrast), presented on a gray background. The numerosity of the reference array was either 8 or 32 dots in Exp. 1, and either 12 or 24 dots in Exp. 2. The probe array had always 16 dots in both experiments. In Exp. 1, the field area (i.e., the area of the virtual circular region over which the dots were drawn) had a diameter of 400 pixels, thus encompassing approximately 9.3° of visual angle. In Exp. 1a, the intervals were marked by a grayscale random-noise circular texture briefly flashed over the dot array for a single screen frame (8 ms), at both the onset and offset of the interval. The texture had the same area of the dot array in order to cover it completely. In Exp. 1b, the intervals were marked by brief “blinks” of the array itself. More specifically, the array disappeared for a single screen frame (8 ms). In doing so, in both Exp. 1a and 1b the array was equally not visible (i.e., either covered by the texture or not presented on the screen) for 8 ms. An example of the stimuli and procedure of Exp. 1 is shown in Figure 1.

In Exp. 2a, the dot arrays could be arranged either in a circular area as in Exp. 1, or in an annular area either contiguous to the area where the interval markers were flashed, or far from it. The circular area used in the “same position” condition had a radius of 150 pixels (3.5°). The annulus used in the “surrounding annulus” condition had an inner radius of 150 pixels (3.5°) and an outer radius of 300 pixels (7°). The annulus used in the “far annulus” condition had an inner radius of 300 pixels (7°) and an outer radius of 400 pixels (9.3°). The dimensions of the two annuli were chosen to keep their area similar (212,000–219,000 pixel^2^). In this experimental condition, the circular texture marking the onset and offset of the interval had a radius of 150 pixels (3.5°). The dot-array stimuli used in Exp. 2b were identical to those used in Exp. 1b, except for their numerosity (12 or 24 dots instead of 8 or 32 dots). In the “whole set” condition, all the dots blinked (i.e., disappeared for 8 ms) to mark the onset and offset of the intervals. In the “subset” condition, only a group of four dots (randomly selected before the presentation of each stimulus) blinked to mark the intervals. In the “red subset” condition, again, only four randomly-selected dots blinked to mark the intervals, but were colored in red from the onset of the dot array to increase their salience and make them more different than the rest of the array. A depiction of the stimuli used in Exp. 2 is shown in Figures 3A–B.

In both experiments, the durations compared by participants were a constant reference interval (always 300 ms), and a variable probe interval (150, 200, 250, 300, 350, 450, or 600 ms). The stimuli could be presented in two different positions, either on the left or on the right of the central fixation point. The horizontal eccentricity of the stimuli (from the center of the stimulus area to center of the screen) was 480 pixels (11.2°). See Figure 1 for a depiction of the stimulus positions. Finally, in all cases each individual dot had a diameter of 20 pixels (0.46°), and the positions of the dots in each array were computed online in a trial-by-trial fashion. Dot positions were constrained only by a minimum inter-dot distance equal to the diameter of a single dot (20 pixels), to avoid overlapping items.

### Experimental design and procedure

The experiment was performed individually in a dimly lit and sound-proof room. In all experiments, participants performed a duration comparison task, comparing the duration of two empty intervals (i.e., intervals marked only at the onset and the offset): a constant reference (300 ms) and a variable probe (150–600 ms) interval. In each trial, while participants kept their gaze on a central fixation point, the reference and probe stimuli were presented sequentially on the screen, with their position (left or right of the fixation point; see Figure 1B) and presentation order (reference first or probe first) randomly determined in a trial-by-trial fashion. The inter-stimulus interval (ISI) between reference and probe was randomly selected from a uniform distribution spanning 650 to 750 ms. Participants were instructed to compare the two temporal intervals and respond by indicating which one of the two was longer. Responses were provided at the end of each trial by pressing either the left of the right arrow on a standard keyboard, indicating the side of the screen where the longer interval was presented. After providing a response, the next trial started automatically after a variable interval of 400 to 600 ms. Feedback about the response was never provided. Before the start of the experiment, each participant performed a few practice trials (5–10 trials). More details specific to each experiment are provided below.

#### Experiment 1

Exp. 1 was divided in two experimental conditions (Exp. 1a and 1b), performed by two (partially) independent groups of participants. In Exp. 1a, we tested the effect of numerosity on perceived duration with these two magnitude dimensions conveyed by distinct stimuli. When the reference was presented first, the trial begun with a dot array appearing on one side on the screen, containing either 8 or 32 dots (reference array). After 500 ms from the onset of the array, the first interval marker was presented. Specifically, a circular texture was flashed for 8 ms covering the entire dot array. The second interval marker (the same texture flashed for 8 ms) was presented after 300 ms (reference duration). After the offset of the second marker, the dot array remained on the screen for 300 ms before disappearing. After an ISI of 650 to 750 ms, a second dot array appeared on the opposite side of the screen, containing 16 dots (probe array). Again, after 500 ms from the array onset, the first marker was presented, followed after an interval ranging from 150 to 600 ms by the second marker (probe duration). The opposite sequence of stimuli was delivered to participants in the case of the probe presented first. At the end of the trial (after the offset of all the stimuli), participants had to indicate which interval lasted longer. Participants were instructed to focus uniquely on the duration of the intervals and ignore the dot arrays.

In Exp. 1b we assessed the bias provided by numerosity on perceived duration with these two dimensions conveyed by the exact same stimulus. The temporal dynamic of Exp. 1b was identical to Exp. 1a, with the exception that no circular texture was presented to mark the intervals. In the case of the reference presented first, the trial started with a dot array appearing on the screen containing either 8 or 32 dots. After 500 ms, the array briefly disappeared (“blinked”) for 8 ms, marking the onset of the reference interval. After 300 ms, the dot array blinked again to mark the offset of the interval. The dot array remained on the screen for 300 ms before disappearing. After the ISI (650–750 ms), the second dot array appeared on the opposite side of the screen, containing 16 dots. After 500 ms the array blinked to mark the onset of the probe interval, and after an interval ranging from 150 to 600 ms blinked again to mark its offset. Again, the dot array remained on the screen for 300 ms, and its disappearance signaled the end of the trial. The opposite sequence of stimuli was followed in the case of the probe presented first. Similarly to Exp. 1a, participants were instructed to compare the duration of the two intervals while ignoring the numerosity of the dot arrays.

Participants performed a total of 280 trials in each experimental condition, including 20 repetitions of each combination of reference numerosity and probe duration. A depiction of the stimuli and procedure of Exp. 1 is shown in Figure 1.

#### Experiment 2

Exp. 2 was similarly divided in two experimental conditions, where we tested the influence of numerosity on perceived duration when they are conveyed by distinct stimuli (Exp. 2a) or by the same stimulus (Exp. 2b). The aim of this experiment was to better assess the properties of the magnitude integration effect in these two cases, and control for possible alternative explanations involving a response bias, a mnemonic rather than a perceptual effect, and the possible role of attention. In both experimental conditions, the procedure was identical to Exp. 1, with a few exceptions detailed below.

In Exp. 2a, we assessed the spatial selectivity of the effect observed when duration and numerosity belong to different stimuli. Testing for the spatial selectivity of this effect was not only aimed at better understanding its properties, but also at clarifying its nature and rule out possible alternative interpretations. Indeed, a genuine perceptual effect that alters the appearance of the stimuli (i.e., their perceived magnitude) is expected to be selective for the position of the stimuli. In other words, a perceptual effect is more likely to arise from the interaction of stimulus representations encoded in a topographic map of the visual field, and should work only when two stimuli are matched in position. On the contrary, a decisional effect like a response bias (i.e., increase in the probability of selecting one response driven by irrelevant information), should work irrespective of the spatial match of the stimuli (e.g., see for instance Quinn, Seillier, Butts, & Nienborg, 2021). In this experiment, three types of trials were randomly intermixed within each block of trials, corresponding to different conditions. The first condition involved flashing the interval markers directly on top of the array, covering its area entirely (“same position”), similarly to Exp. 1a. The second condition involved arranging the dot array in an annulus spatially surrounding the location of the interval markers (“surrounding annulus”). The third condition involved again arranging the dot array in an annulus, but presenting it spatially displaced from the location of the interval markers (i.e., 100 pixels [2.3°] from the outer border of the markers to the inner border of the annulus). With the exception of the position and shape of the dot arrays, all the other parameters of the experiment were identical to Exp. 1a.

In Exp. 2b we focused instead on the effect observed in the condition where duration and numerosity are conveyed by the same stimulus, and we assessed whether having the whole stimulus (i.e., all the dots in the array) conveying the duration information is a necessary condition to observe the integration effect. According to the idea of a magnitude binding, the integration effect should be observed only when both dimensions are coherently conveyed by the stimulus in its entirety. For example, the effect that a numerosity of 24 dots has on duration should be observed only when all the 24 dots blink to mark the interval duration. However, alternative interpretations of the effect and the difference between different cases (i.e., magnitudes conveyed by a single stimulus vs. two separate stimuli) include the possibility of a mnemonic effect, and a critical role of attention. Indeed, first, the magnitude integration bias could be explained by a mnemonic (rather than perceptual) interference (Cai et al., 2018) caused by the encoding of the irrelevant dimension of the stimulus (i.e., numerosity) in working memory (e.g., Bocincova & Johnson, 2019). Second, any difference in the effect measured in different cases (Exp. 1a vs. 1b) might be explained by attention being directed to the dots as opposed to attending the duration markers (i.e., the texture) and ignoring the dots. In these cases, whether the entire stimulus or only a subset of it is used to mark the interval duration should not affect the interference, and a bias should always be observed. This experiment was similarly composed of three different trial types (conditions), intermixed within each block. In one condition (“whole set”), the onset and offset of the intervals was signaled by a blink of the entire dot array, as in Exp. 1b. In the second condition (“subset”), the onset and offset of the intervals was signaled by a blink of only four dots. The subset of four dots blinking was randomly selected before the presentation of the stimulus. Finally, in the third condition (“red subset”), the interval duration was similarly marked by a blink of a randomly-selected subset of four dots, but this time they were colored in red starting from the onset of the array. With the exception of these conditions, all the other aspects of the experiment were identical to Exp. 1b. Importantly, as mentioned above, the “subset” condition also served as a control for the possible role of attention in determining the effect provided by numerosity. Namely, even if only a subset of the array blinked to mark the interval, the randomness of the subset selection forced participants to attend the entire array, allowing to assess whether this is a sufficient condition to induce an effect based on the stimulus numerosity.

In both Exp. 2a and 2b, participants performed a total of 840 trials, including 20 repetitions of each combination of condition, reference numerosity, and probe duration. A depiction of the stimuli used in Exp. 2 is provided in Figures 3A–B.

### Data analysis

**Figure 2.**
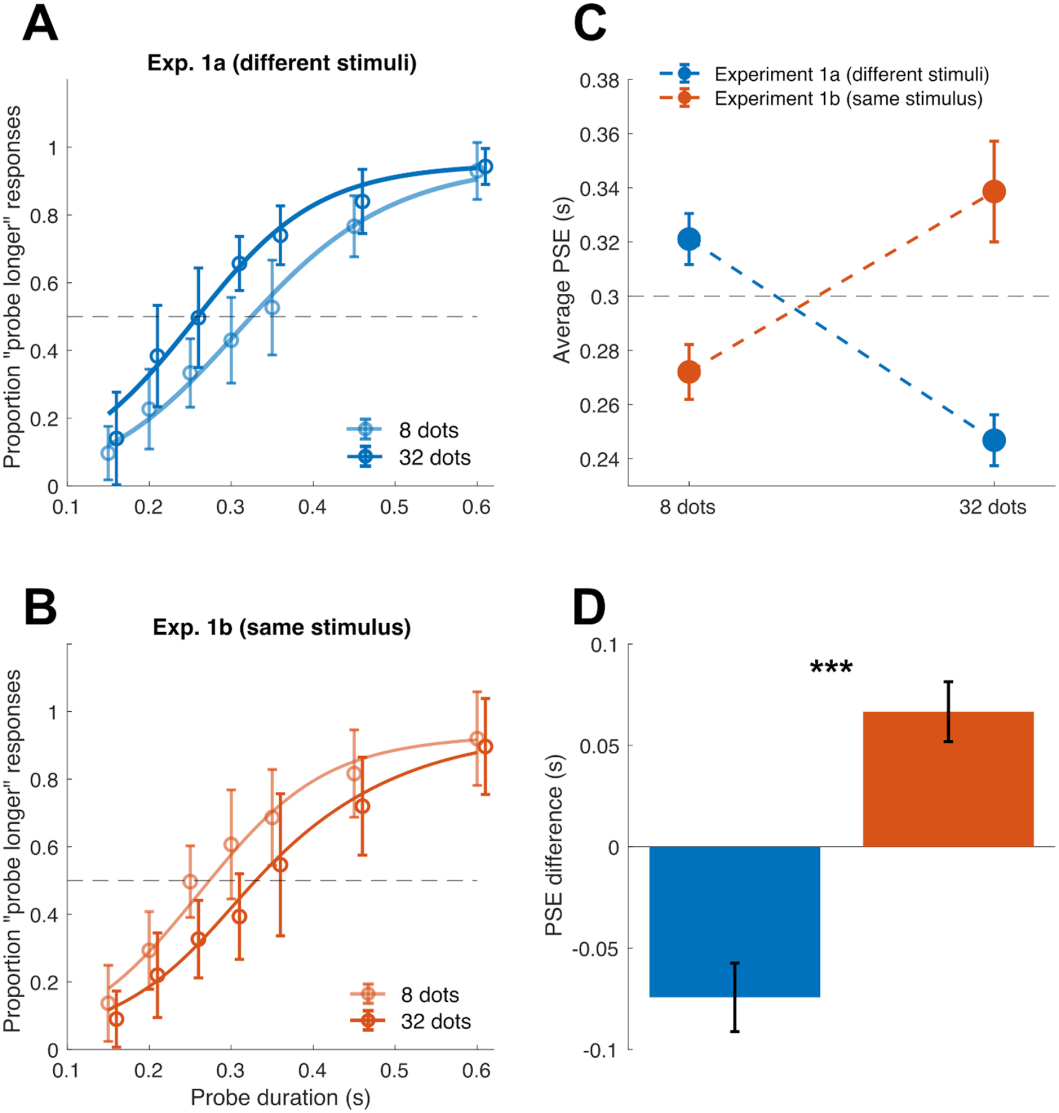
Results of Exp. 1. (A) Average psychometric curves in Exp. 1a, plotted according to the numerosity of the reference dot array (8 or 32 dots). (B) Average psychometric curves in Exp. 1b. The *dashed line* indicates chance level responses. *Error bars* represent one standard deviation. (C) Average PSEs measured in Exp. 1a and 1b, as a function of the reference numerosity. The *horizontal dashed line* indicates the objective duration of the reference (300 ms). (D) Average difference in PSE (PSE obtained with 32 dots minus PSE obtained with 8 dots) measured in Exp. 1a and 1b. A negative PSE difference indicates that perceived duration decreases as numerosity increases, whereas a positive difference indicates a positive relation between numerosity and perceived duration. ***p < 0.001 (independent samples *t*-test across experiments). *Error bars* are SEM.

Across all experiments, the effect of the different experimental manipulations was assessed in terms of difference in the point of subjective equality (PSE), which reflects the accuracy in the task and the perceived duration of the reference stimulus. For each participant and condition, the proportion of “probe longer” responses across the different levels of probe duration was fitted with a cumulative Gaussian function, according to the maximum likelihood method (Watson, 1979). The average psychometric curves are shown in Figures 2A–B (Exp. 1) and Figure 4 (Exp. 2). Within each participant and experimental condition, the fitting procedure was performed separately according to the numerosity of the reference array, to assess the perceived duration of the reference as a function of numerosity. The PSE was defined as the median of the cumulative Gaussian fit. As a measure of precision in the task, we first computed the just noticeable difference (JND), as the difference in probe duration between chance level responses, and 75% “probe longer” responses. The JND was then used to compute the Weber's fraction (WF = JND/PSE) as an additional measure of precision in the task. Measures of the precision in the task are shown in Supplementary Figure S2 (Exp. 1) and S4 (Exp. 2). To exclude participants showing insufficient performance, we set an exclusion threshold at WF ≥ 1. One participant in Exp. 2 was excluded from data analysis due to excessively poor performance, according to this exclusion criterion.

Moreover, to better appreciate the strength and direction of the magnitude integration effect, in both Exp. 1 and Exp. 2 we computed the difference in PSE as a function of the numerosity displayed:

(1) $$\mathrm{PSE}\;\mathrm{difference}=\mathrm{PS}E_{\mathrm{high}}-\mathrm{PS}E_{\mathrm{low}};$$

Where PSE_high_ indicates the PSE obtained when the reference had a higher number of dots compared to the probe (32 or 24, respectively for Exp. 1 and Exp. 2), and PSE_low_ indicates the PSE obtained when the reference had a lower number of dots compared to the probe (8 or 12, respectively for Exp. 1 and Exp. 2). A negative PSE difference indicates a repulsive effect, whereby perceived duration gets shorter with increasing numerosity, while a positive difference indicates an attractive, congruent bias, with perceived duration increasing as the numerosity increases.

To assess the significance of the biases in perceived duration, in Exp. 1 we first performed a two-way independent-samples analysis of variance (ANOVA) on PSE values, with factors “numerosity” (8 vs. 32), and “experiment” (Exp. 1a vs. Exp. 1b). The ANOVA was followed up with two paired *t*-tests (considering a Bonferroni-corrected alpha of 0.025) comparing the PSEs corresponding to the different numerosities within each experiment. Finally, we compared the effect measured in the two experiments with an independent-samples *t*-test performed of the PSE difference measure. Exp. 2a and 2b were instead analyzed separately, employing a two-way repeated measures ANOVA with factors “numerosity” and “condition.” We again followed up the ANOVA with a series of paired t-tests comparing the PSEs within each condition (Bonferroni-corrected alpha = 0.017), and, finally, with two paired *t*-tests (alpha = 0.025) comparing the PSE difference in the “same position” condition against the “surrounding annulus” and the “far annulus” condition (Exp. 2a), or the “whole set” condition against the “subset” and “red subset” condition.

### Data availability

All the data generated during the experiments described in this manuscript can be found on Open Science Framework following this link: https://osf.io/gt5cx/. Experimental code will be made available by the corresponding author on request.

## Results

### Magnitude “binding” or contextual interference?

In Exp. 1, two groups of 15 participants performed a duration comparison task. comparing the duration of a constant reference stimulus (300 ms) with a variable probe interval (ranging from 150 to 600 ms). To bias duration perception, the numerosity of the reference varied from trial to trial (either 8 or 32 dots) whereas that of the probe was kept constant (16 dots). Numerosity was task irrelevant. Participants performed this task in two distinct stimulation conditions (see Figure 1). In Exp. 1a, duration and numerosity were conveyed by two distinct stimuli. Each temporal interval was defined by two briefly-presented (8 ms) circular textures marking the onset and offset of the interval, flashed on top of the dot array conveying the task-irrelevant numerosity. In Exp. 1b, instead, duration and numerosity were conveyed by the exact same stimulus: the onset and offset of the intervals were marked by brief blinks of the dot array itself (i.e., disappearing for 8 ms). In general, what we expected in terms of magnitude integration was a relative under- or over-estimation of the reference duration compared to the probe stimulus induced by the numerosity, when the reference was coupled with either fewer (8) or more dots (32) compared to the probe (16 dots).

The perceived duration of the reference stimulus was assessed in terms of the point of subjective equality (PSE), derived from a cumulative Gaussian fit (Watson, 1979) (i.e., psychometric curve) applied to the proportion of “probe longer” responses (see Methods). Figures 2A and 2B show the average psychometric curves in Exp. 1a and 1b, respectively, as a function of the numerosity of the reference dot array. In both cases we observed a marked difference in the psychometric curves, but, strikingly, in two opposite directions. Namely, while increasing numerosity caused a leftward shift of the curve in Exp. 1a (i.e., reflecting an underestimation of the reference duration), in Exp. 1b the shift was rightward (i.e., overestimation of the reference duration with increasing numerosity).

Figure 2C reports the average PSEs as a function of numerosity. First, a two-way independent samples ANOVA on PSE values, with factors “numerosity” (8 vs. 32 dots), and “experiment” (Exp. 1a vs. 1b), showed neither a significant effect of numerosity (*F*(1,56) = 0.09, *p* = 0.760), nor a significant effect of experiment (*F*(1,56) = 2.92, *p* = 0.093). Most importantly, however, we observed a significant interaction between the two factors (*F*(1,56)= 31.72, *p* < 0.001, *η*^2^_p_ = 0.36), suggesting an opposite relationship between numerosity and perceived duration across the two conditions.

We followed up the interaction by running two paired t-tests (Bonferroni-corrected alpha = 0.025), comparing the PSE obtained with different numerosities within each experiment. The results showed a significant difference in both cases, but in opposite directions (t(14) = 4.40, *p* < 0.001, Cohen's *d* = 1.14; *t*(14) = −4.51, *p* < 0.001, *d* = 1.17).

To better appreciate the extent of the bias and its direction in the two experiments, we computed the difference in the PSE obtained with different numerosities (Figure 2D). This difference showed robust biases in perceived duration in both cases (mean ± *SD*: 74 ± 65 ms and 66 ± 57 ms, respectively for Exp. 1a and 1b), but, again, in opposite directions. Although the effect observed in Exp. 1b is in line with a congruent magnitude integration bias (i.e., the higher the numerosity, the longer the perceived duration of the reference), the effect in Exp. 1a is an opposite, “repulsive” bias (i.e., the higher the numerosity, the shorter the perceived duration). Comparing the PSE difference across the two experiments confirmed that the effects are significantly different from each other (independent-samples *t*-test, t(28) = −6.28, *p* < 0.001, *d* = 2.29). Individual measures of PSE are shown in Supplementary Figure S1, whereas Supplementary Figure S2 shows the participants’ precision in the task (Weber's fraction [WF]; see Supplementary Materials).

### Selectivity and properties of magnitude integration

In Exp. 2, we further addressed the properties of the two opposite effects found in Exp. 1. In this context, we had two main questions. First, does the repulsive effect observed in Exp. 1a require a spatial correspondence between numerosity and duration information (i.e., stimuli located in the same spatial position; spatial selectivity), or does the effect work even if numerosity is presented anywhere in the visual field? Second, does the attractive magnitude integration bias observed in Exp. 1b require the whole stimulus to convey duration information, or only a part of the object (i.e., a subset of dots) is sufficient to establish the integration? In other words, does the integration effect always work according to the overall numerosity of the array, or according to the numerosity of the dots effectively conveying duration information?

**Figure 3.**
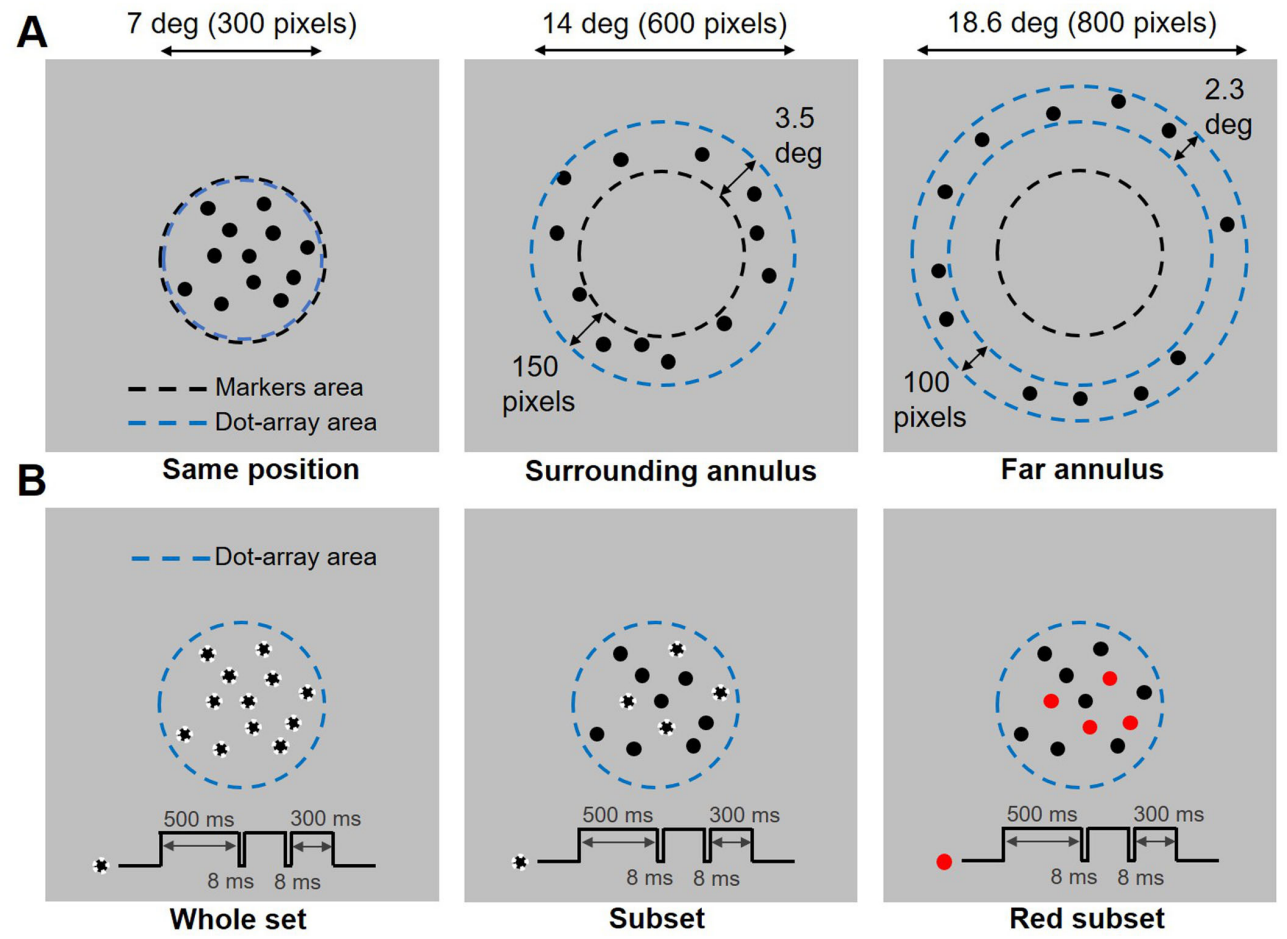
Stimuli used in Exp. 2. (A) The different stimulation conditions tested in Exp. 2a included (from left to right): a condition where the area of the interval markers corresponded to the dot array (“same position”), a condition in which the dot array was structured as an annulus surrounding the area where the interval markers were flashed (“surrounding annulus”), and finally a condition in which the dot array, structured again as an annulus, was presented far away from the interval markers (“far annulus”). These three conditions were randomly intermixed within the same blocks of trials. (B) In Exp. 2b, we tested a condition in which all the dots in the array blinked to mark the interval (“whole set”), a condition in which only a subset of four dots blinked to mark the intervals (“subset”), and finally a condition in which a subset of four dots colored in red blinked to mark the intervals (“red subset”). Again, these conditions were intermixed within the same blocks of trials. Stimuli are not depicted in scale, and dots in the actual experiment were black and white in equal proportions.

In Exp. 2, a group of 19 participants was tested in two distinct experimental conditions investigating the properties of the repulsive (Exp. 2a) and attractive (Exp. 2b) biases observed before. First, we assessed the spatial selectivity of the repulsive effect, using a methodology identical to Exp. 1a with only a main difference: we changed the spatial position of the dot array with respect to the position of the circular texture marking the temporal intervals (Figure 3A). In different trials, the dot array could be presented in the same spatial location of the interval markers (similar to Exp. 1a; “same position”), or arranged in an annulus either near (“surrounding annulus”) or far from the markers (“far annulus”). The rationale for these three conditions was to assess the selectivity and tolerance of the effect for the spatial correspondence of the magnitudes conveyed by the dots (numerosity) and by the texture markers (duration). If the effect is perceptual in nature (i.e., related to perceptual processing in brain areas representing a topographic map of the external world), then the effect is expected to be selective for the relative position of the stimuli, and should emerge only when the stimuli are presented in the same position. Conversely, if the effect is more akin to a response bias (i.e., change in the probability of response due to the presence of irrelevant information), then such a selectivity is expected to be weaker or absent. In Exp. 2b, on the other hand, we assessed whether the attractive effect observed in Exp. 1b requires the whole stimulus, or only a part of it (i.e., a subset of dots; Figure 3B). In this experiment, the temporal interval could be marked by the blinks of either the whole array (i.e., as in Exp. 1b; “whole set”), a random subset of four dots of the array (“subset”), or a subset of four dots displayed in a different color (“red subset”). Importantly, the subset condition also allowed to assess whether attention could play a role in determining the effect. Indeed, since the subset was randomly chosen before the onset of each array—making it unpredictable—participants were forced to attend the entire array. On the other hand, the red subset condition was added to test an additional prediction: if the red subset is treated as a distinct object (like in Exp.1a), then we may observe a repulsive bias as we did in Exp.1a. Differently from Exp.1, in both Exp. 2a and 2b the numerosity of the dot array corresponding to the reference interval was either 12 or 24 dots, whereas the array corresponding to the probe always contained 16 dots.

**Figure 4.**
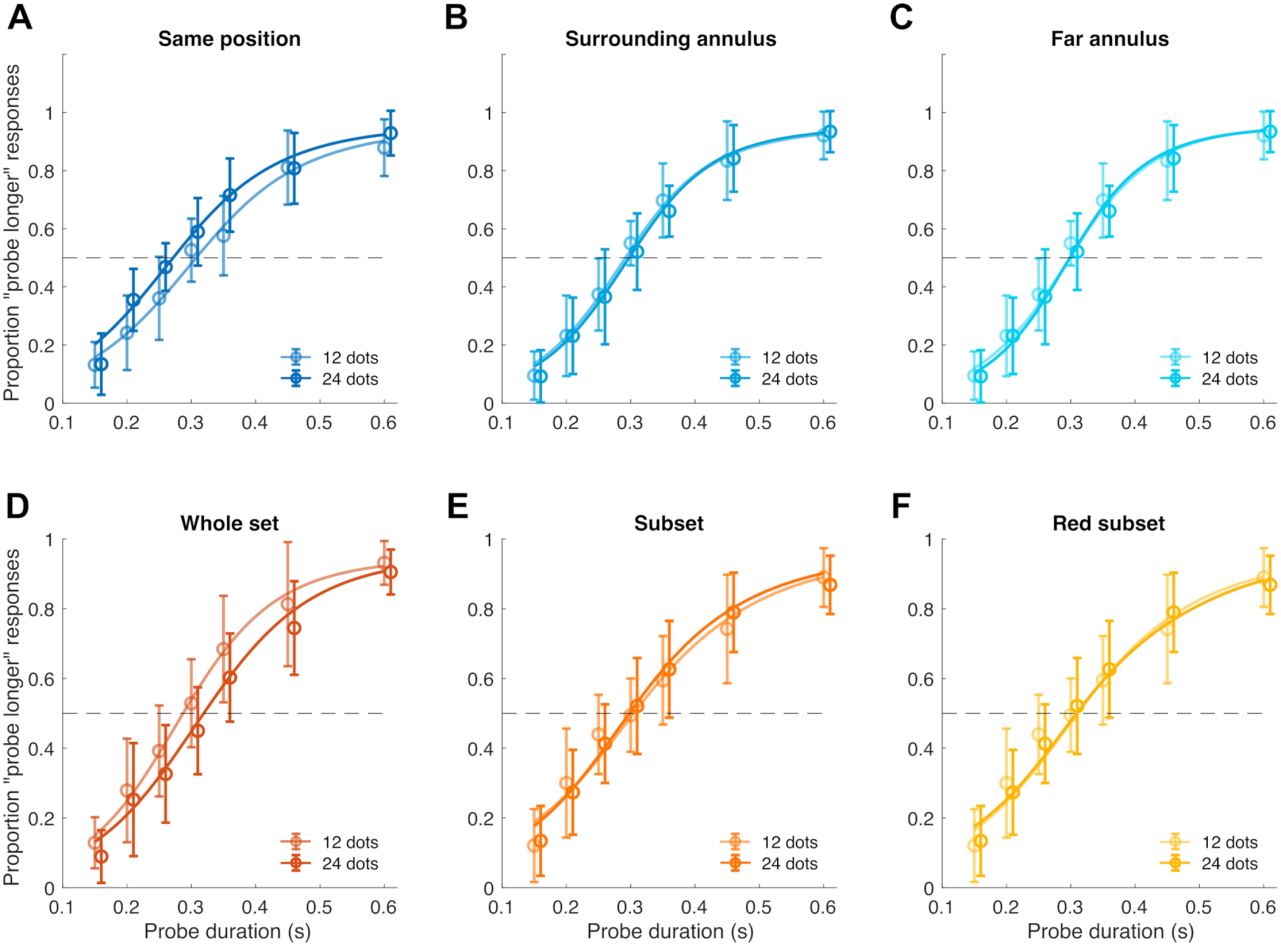
Average psychometric curves obtained in the different conditions of Exp. 2. (A) Psychometric curves corresponding to the two numerosities associated with the reference presentation (12 and 24 dots), in the “same position” condition of Exp. 2a. (B) Psychometric curves in the “surrounding annulus” condition of Exp. 2a. (C) Psychometric curves in the “far annulus” condition of Exp. 2a. (D) Psychometric curves in the “whole set” condition of Exp. 2b. (E) Psychometric curves in the “subset” condition of Exp. 2b. (F) Psychometric curves in the “red subset” condition of Exp. 2b. *Error bars* represent one standard deviation.

Figure 4 shows the average psychometric curves in the different conditions of Exp. 2a (A-C) and Exp. 2b (D-F), corresponding to the two numerosities tested. Similarly to Exp. 1a, in Exp. 2a we observed a noticeable leftward shift in the psychometric curve as the numerosity increased, suggesting a “repulsive” effect whereby higher numerosity is associated with shorter perceived duration. However, this effect was observed only when the dot array and the interval markers were matched in space (same position condition, Figure 4A). When the dots were displaced from the location of the interval markers (Figures 4B, 4C), the psychometric curves observed are virtually identical, suggesting no difference in perceived duration. In Exp. 2b we instead observed a rightward shift in the psychometric curves when the entire array blinked to mark the interval (whole set condition, Figure 4D), showing an increase in perceived duration with increasing numerosity. When only a subset of the array blinked to mark the interval (Figures 4E, 4F), no difference in the psychometric curves was observed, suggesting again no difference in perceived duration.

**Figure 5.**
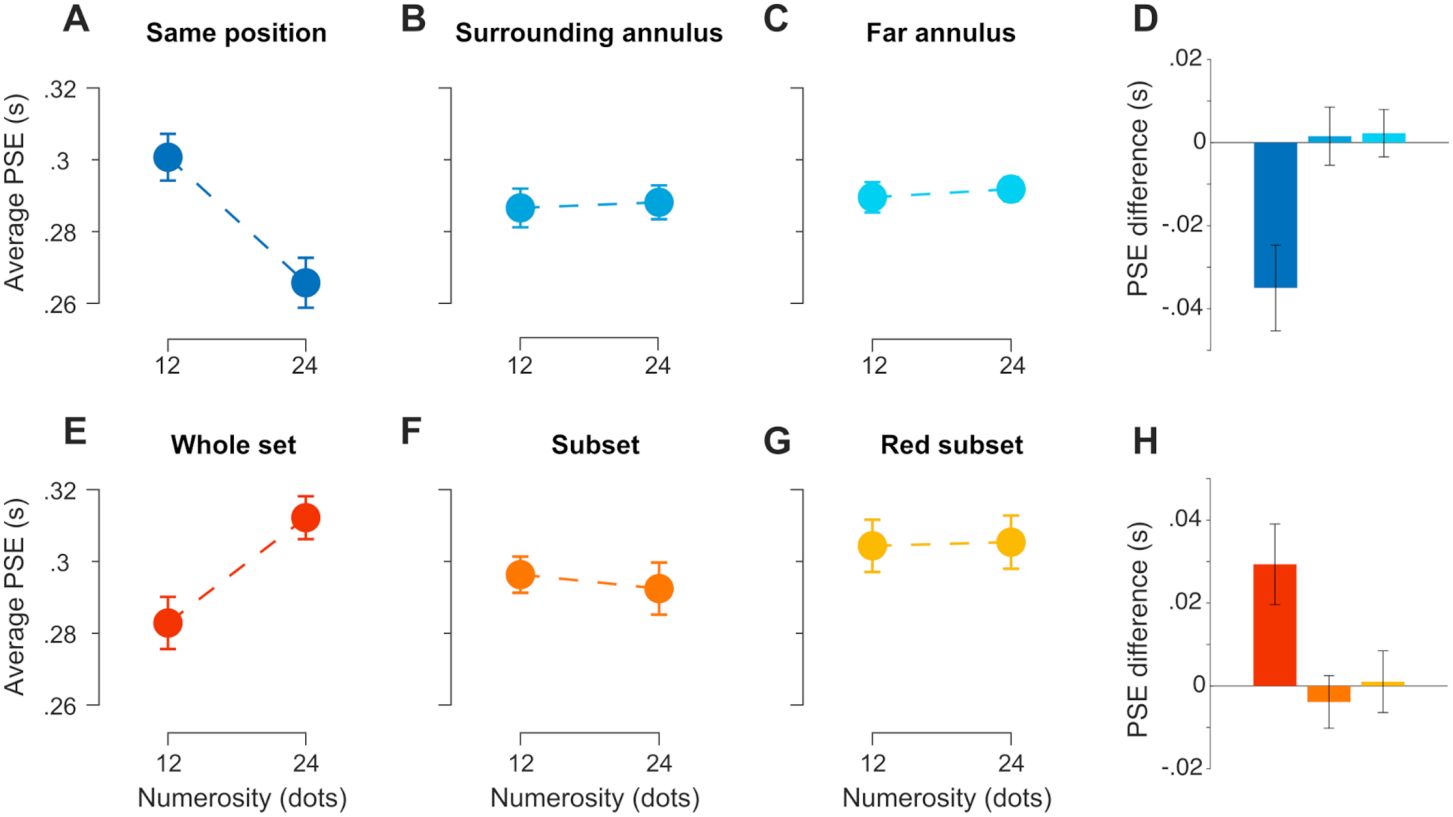
Results of Exp. 2. (A-D) Results of Exp. 2a. (A) Average PSEs as a function of numerosity, when the interval markers were presented on top of a dot array with either a low (12 dots) or high (24 dots) numerosity. (B) Average PSEs obtained when arranging the dot array in an annulus surrounding (but not overlapping) the position of the interval markers. (C) Average PSEs when the dot-array annulus was positioned away from the location of the markers. (D) Summary of results obtained in Exp. 2a, in terms of the difference in PSE obtained with different numerosities. (E–H) Results of Exp. 2b. (E) Average PSEs as a function of numerosity when the intervals were marked by blinks of the whole dot array. (F) Average PSEs when only a subset of the array blinked to mark the intervals. (G) Average PSEs when the subset of dots blinking to mark the interval was presented in red. (H) Summary of the effects observed in Exp. 2b, in terms of PSE difference. *Error bars* are SEM.

In Exp. 2a, the results in terms of average PSE confirm indeed a strong repulsive effect when presenting the dot array in the same spatial position of the interval markers (Figure 5A), replicating the results of Exp. 1a. When the dots were instead positioned in an annulus surrounding the markers (Figure5B) or far away from it (Figure 5C), little or no effect was observed. To better appreciate the strength and direction of the effect, we computed again the difference in PSE, which is shown in Figure 5D. A two-way repeated measures ANOVA on PSEs, with factors “numerosity” (12 vs. 24 dots) and “condition” (same position, surrounding annulus, far annulus), showed a significant main effect of numerosity (*F*(1,18) = 6.65, *p* = 0.019, *η*^2^_p_ = 0.27), no significant effect of condition (*F*(2,36) = 1.11, *p* = 0.340), and a significant interaction between the two factors (*F*(2,36) = 6.51, *p* = 0.004, *η*^2^_p_ = 0.26). We followed up this interaction by first running a series of (Bonferroni-corrected) paired *t*-tests (alpha = 0.017) within each condition. In the same position condition, we observed a statistically significant difference in PSE according to numerosity (*t*(18) = 3.39, *p* = 0.003, *d* = 0.78). Conversely, no significant difference was observed in the surrounding and far annulus condition (*t*(18) = −0.22, *p* = 0.826, *d* = 0.05, and *t*(18) = −0.40, *p* = 0.695, *d* = 0.09). Finally, we used the difference in PSE (Figure 5D) to compare the same position condition against the other two (Bonferroni-corrected alpha = 0.025). The results showed a significant difference both when comparing the same position versus the surrounding annulus condition (*t*(18) = 2.67, *p* = 0.015, *d* = 0.66), and the same position versus the far annulus condition (*t*(18) = 3.01, *p* = 0.007, *d* = 0.69).

In Exp. 2b, the results in terms of average PSE first replicated the results of Exp. 1b (Figure 5E), by showing an attractive bias of numerosity on duration perception (i.e., the more numerous the longer) when numerosity and duration are conveyed by the same object. Conversely, with only a subset of dots of either the same or different color marking the intervals, we did not observe substantial biases (Figures 5F, 5G). To assess the pattern of results, we again used a two-way repeated measures ANOVA with factors “numerosity” and “condition” (whole set, subset, red subset), as in Exp. 2a. The results showed a significant main effect of numerosity (*F*(1,18) = 6.96, *p* = 0.017, *η*^2^_p_ = 0.28), a significant effect of condition (*F*(2,36) = 3.32, *p* = 0.048, *η*^2^_p_ = 0.16), and a significant interaction between the two factors (*F*(2,36)= 4.11, *p* = 0.025, *η*^2^_p_ = 0.19). A series of paired *t*-tests (alpha = 0.017) following up the interaction further showed a significant difference in PSE according to numerosity in the whole set condition (*t*(18) = 3.02, *p* = 0.007, *d* = 0.69), and no significant differences in the subset (*t*(18) = −0.61, *p* = 0.548, *d* = 0.14) and red subset condition (*t*(18) = 0.14, *p* = 0.893, *d* = 0.03). Finally, we compared the PSE difference in the whole set condition against the other two conditions (alpha = 0.025). These tests showed a significant difference between the whole set and subset condition (*t*(18) = 2.53, *p* = 0.021, *d* = 0.58), but not between the whole set and the red subset condition (*t*(18) = 2.07, *p* = 0.054, *d* = 0.47), although this comparison still showed a medium effect size. Individual measures of PSE and of the WF are shown in Supplementary Figures S3 and S4.

## Discussion

The current study addresses a fundamental question about the nature of magnitude integration and perception: does magnitude integration involve a “binding” of the different dimensions of the same object, or does it represent just a contextual interference working irrespective of the object the different magnitudes belong to? The bias induced by the integration of different magnitude dimensions (Cappelletti et al., 2009; Javadi & Aichelburg, 2012; Xuan et al., 2007) is indeed an important process defining how we perceive and judge magnitudes. Still, the nature of this process remains unclear. Here we show for the first time that magnitude integration is not a passive contextual or mnemonic interference (Cai et al., 2018) but most likely involves an active binding process concerning the different dimensions of the same object. The attractive bias defining a congruent magnitude integration (e.g., the more numerous a set of items is, the longer it appears to last in time; Javadi & Aichelburg, 2012) was indeed observed only when the two magnitude dimensions are conveyed by the exact same stimulus (Exp. 1a). When two dimensions are conveyed by two clearly distinct stimuli, perceived duration was repulsed away from numerosity, yielding an opposite effect (Exp. 1b).

Moreover, results from Exp. 2a show that the repulsive effect is strictly spatially localized to the position of the stimuli: the effect emerges only when the interval markers are overlapping with the dot array. If instead the dot array surrounds the location of the interval markers, we observed no effect. This suggests that the bias provided by one stimulus to the perceived magnitude of another one is not a decisional effect like a response bias, but it is perceptual in nature. Indeed, an alternative explanation of the interference across magnitudes is an increase in the probability of selecting a specific response as a function of the irrelevant information provided concurrently to the relevant stimulus. For instance, the mere fact of seeing a large number of dots during the reference presentation (compared to the probe presentation) may simply increase the probability of selecting the “reference more” response, and vice versa when the dots are fewer than the probe. However, such a response bias would be expected irrespective of whether the stimuli occupy the same position or not (e.g., Quinn et al., 2021). It is therefore more likely that the tight spatial selectivity observed in our experiment reflects a magnitude integration effect related to perceptual processing in topographic maps of the visual field (Burr, Tozzi, & Morrone, 2007).

Results from Exp. 2b further show that the congruent, attractive integration bias occurs only if the whole stimulus conveys duration information. If only a subset of the dot array marks the interval, the bias does not emerge. This suggests that magnitude integration is driven by the number of dots effectively conveying duration information, and not necessarily by the entire dot array itself. In this case, however, in the absence of an attractive bias, one might have expected an opposite repulsive effect. Indeed, if the blinking subgroup and the rest of the array are treated as two distinct stimuli, we could have observed the same repulsive effect as in Exp. 1a and 2a. To make the subgroup even more salient and different than the rest of the array, we also presented it in red. Still, no effect was observed. This last set of results demonstrates the importance of the spatial congruence of magnitude dimensions for magnitude repulsion to occur. In other words, even if two distinct sets are intermixed with each other, in the absence of a spatial overlap no effect could be observed (i.e., because the dots in the subset and the rest of the array occupied nearby but non-overlapping positions). Additionally, the results of Exp. 2a and 2b rule out an attentional explanation of the effect. Indeed, first, one could argue that the congruent integration effect might emerge as a result of participants actively attending the dot array, while the opposite repulsive effect would arise from the inhibition of the irrelevant distracting numerosity when participants attended the texture interval markers. However, this possibility is less likely because in the “subset” condition of Exp. 2b, due to fact that all experimental conditions were interleaved and the blinking subsets were randomly chosen, it was impossible for the participants to predict which subset of dots to attend. Thus, although participants had still to attend the whole array to perform the task, no effect was observed in this condition, suggesting that attention per se is unlikely to explain the results. Irrespective from this, the inhibition of numerosity information when attending the interval markers in Exp. 1a/2a would predict an abolished, rather than reversed, effect, and a degradation of performance (i.e., lower precision) due to the additional resources needed for inhibition, which was not observed (see Supplementary Materials). Again, this makes such attentional explanation of the results unlikely. Alternatively, the fact that participants had to potentially divide their attention between the dots and the texture (although not required by the task), might somehow explain the difference in the effect (i.e., underestimation due to less attentional resources dedicated to the interval duration). However, if attention is divided across the two stimuli, it would similarly (or perhaps even more) be divided when the texture did not match the spatial position of the dots. Nevertheless, no effect was observed in such conditions, ruling out a possible influence of divided attention per se. Similarly to the attentional explanation, the results of the subset condition of Exp. 2b also rule out the possibility of a working memory interference. Indeed, one possible explanation for the magnitude integration effect is an interference between magnitude representations concurrently stored in working memory (Cai et al., 2018)*.* For instance, it has been shown that working memory encoding incorporates the irrelevant dimensions of a target stimulus (Bocincova & Johnson, 2019), and this may interfere with the task-relevant dimension during decision-making. However, differently from our findings, this type of working memory interference should work for the entire array irrespective of whether only a subset of items blinks to mark the interval.

Moreover, another potential alternative explanation of the results might involve the amount of “change” occurring in the stimuli at the onset and offset of the interval. Dynamic changes in the stimuli, such as flicker or motion, can indeed affect the perceived duration or numerosity of a stimulus (e.g., Brown, 1995; Fornaciai & Park, 2017; Kanai, Paffen, Hogendoorn, & Verstraten, 2006). According to this idea, in Exp. 1b/2b the higher the number of dots, the higher the amount of change in the stimuli, which may thus lead to an overestimation that would not be strictly related to the integration of duration and numerosity. Conversely, when the texture is flashed over the stimuli (Exp. 1a/2a), the presence of more dots could instead reduce the amount of change, leading to an opposite pattern. This explanation is however unlikely for two main reasons. First, previous results show that the extent to which a dynamic change in the stimuli affects perceived magnitude depends on the duration and frequency of the modulation (Fornaciai & Park, 2017; Kanai et al., 2006). Considering the strength of these effects measured in previous studies, an 8-ms blink of the stimuli is unlikely to yield a 20% change in perceived duration. With such a brief stimulation, we would only expect a bias of a few milliseconds, if any. Second, the amount of change cannot explain the repulsive effect observed in Exp. 1a/2a, because when the texture is flashed over the dots a “change” occurs at almost every pixel of the image irrespective of numerosity. Considering the random nature of the noise texture and that the dots were black and white, increasing the numerosity does not increase the amount of change in luminance compared to the gray background, as the amount of black texture pixels falling over black dots is compensate by the presence of white pixels, and vice versa (i.e., the average change in luminance is identical irrespective of numerosity). A genuine difference in the magnitude integration effect thus remains the most plausible explanation of the results.

Overall, our results show that the congruent integration (i.e., the more numerous the longer) of different magnitudes relies on a binding process linking different dimensions of the same object. This integration might for instance reflect the influence of a “prior” (Kersten, Mamassian, & Yuille, 2004; Knill & Richards, 1996) acquired according to the statistics of the environment, where bigger objects or more numerous groups likely tend to remain visible for a longer time, whereas smaller objects or less numerous groups might inherently entail briefer events. Magnitude integration might thus represent a way to increase the efficiency of brain processing by exploiting expectations and potentially redundant information. When information is conveyed by distinct stimuli in close spatial proximity, the brain may instead try to actively disambiguate them by repulsing the perception of one magnitude away from the other. Similarly to perceptual adaptation (Kohn, 2007), this could for instance serve to de-correlate different representations (Mattar, Olkkonen, Epstein, & Aguirre, 2018) and increase the sensitivity to change (Bex, Bedingham, & Hammett, 1999; Gepshtein, Lesmes, & Albright, 2013; Kristjánsson, 2001). Overall, such a binding process might thus be aimed at solving the same “binding problem” concerning the more general case of object perception, coordinating and linking the representation of different magnitude dimensions and determining the direction of the integration effect. Interestingly, the fact that a congruent magnitude integration is observed only between dimensions of the same object might explain the lack of a common neural “quantity” code for different magnitudes observed in previous studies (Borghesani et al., 2019). Indeed, in the study by Borghesani and colleagues (Borghesani et al., 2019), for instance, different magnitude dimensions were modulated independently in different sessions of the experiment, using different stimuli. This raises the interesting prediction that a common neural quantity code might indeed exist but would only be shared by the different magnitudes of the same stimulus, allowing us to distinguish the representations of distinct objects. In other words, the common quantity code would be created in the process of binding different dimensions in a unified percept.

## Conclusion

To conclude, our results provide for the first time critical evidence concerning the nature of magnitude integration. We show that the congruent integration often observed across magnitude dimensions like numerosity and duration requires them to belong to the same object. This in turn suggests that this effect is not a passive interference between different types of information, but requires an active binding process creating a unified stimulus representation.

## Supplementary Material

Supplement 1
