# Supplementary material for: The nature of magnitude integration: Contextual interference versus active magnitude binding
Source: J Vis. 2022 Oct 19;22(11):11. doi: 10.1167/jov.22.11.11 (PMC9587468; doi:10.1167/jov.22.11.11)
Supplement: Supplement 1 [file jovi-22-11-11_s001.docx]

**SUPPLEMENTARY MATERIALS**


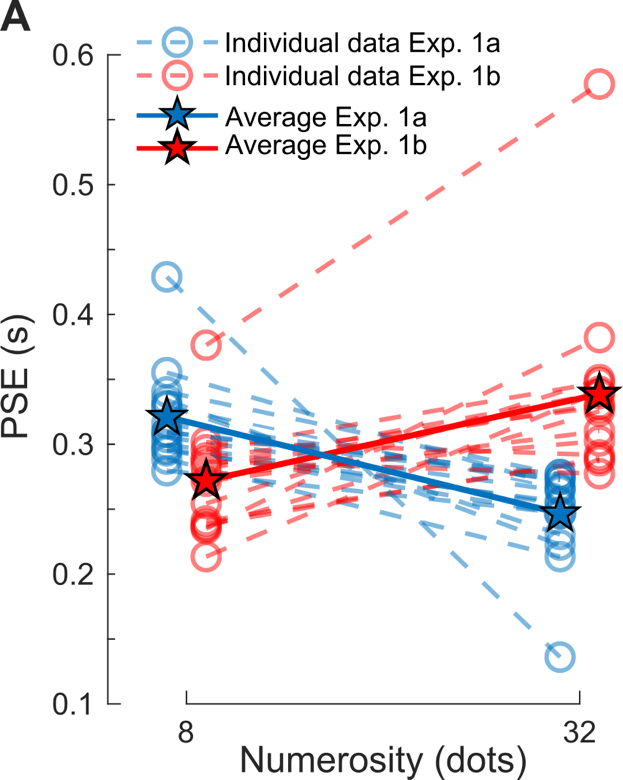


***Figure S1 – Individual data of Exp. 1.*** *Individual PSE (point of subjective equality) values in Exp. 1a (blue circles) and Exp. 1b (red circles) as a function of reference numerosity. The average PSEs in the different experimental conditions are marked with stars. The PSE reflects the participant’s accuracy in the duration comparison task, and the perceived duration of the reference stimulus. In Exp. 1a, all the participants showed the repulsive effect induced by numerosity on the perceived duration of the reference, while in Exp. 1b 14 out of 15 participants showed a congruent effect of numerosity on perceived duration.*


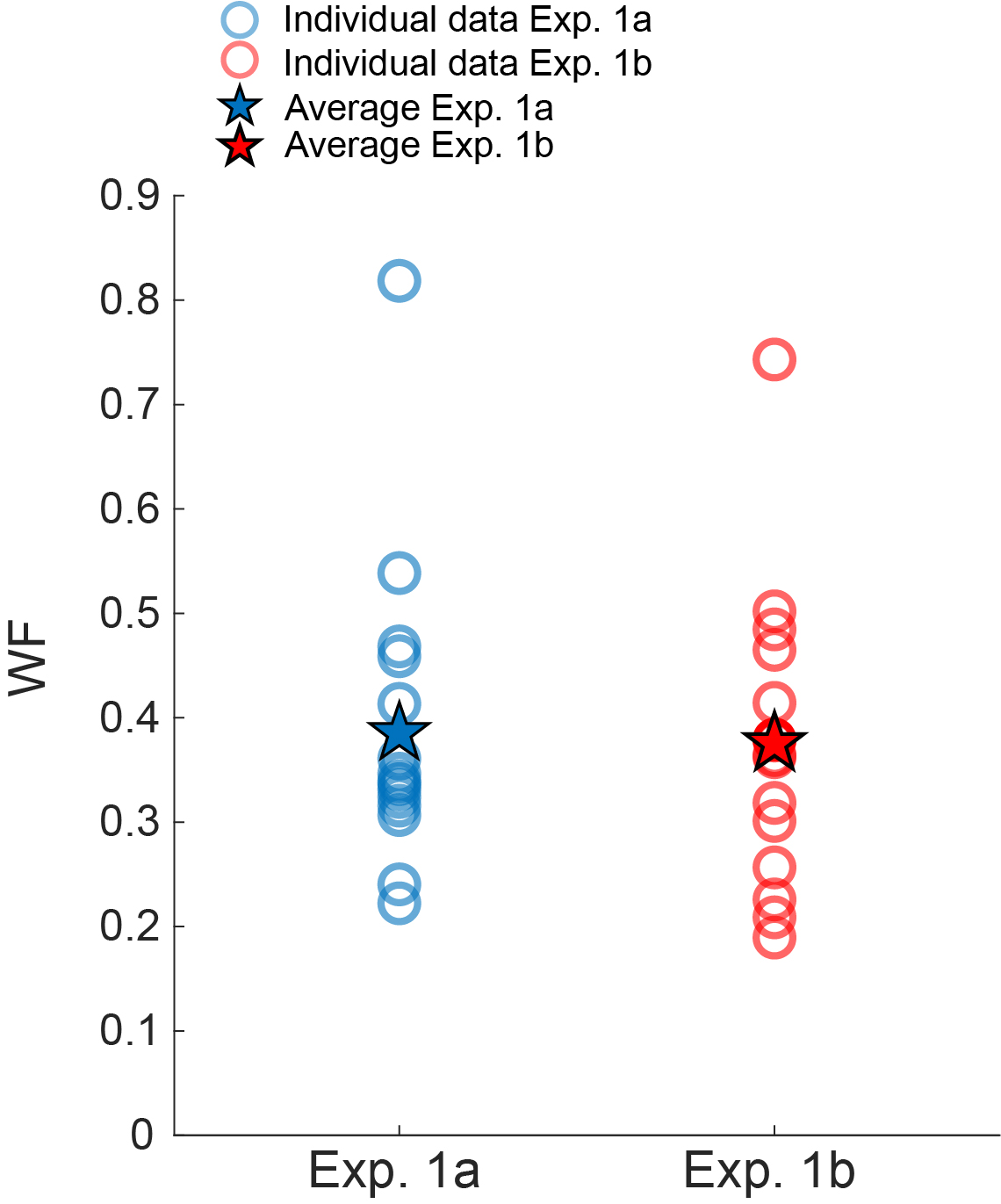


***Figure S2 – Weber’s fractions (WF) in Exp. 1****. The figure shows the individual WFs in Exp. 1a and Exp. 1b (blue and red circles, respectively), averaged across the two levels of reference numerosity, as well as the average across the two group of participants (blue and red star). The WF reflects the precision in the task, and the participants’ sensitivity to duration.*

As a measure of precision in the task, we analysed the Weber’s fraction (WF), computed as JND/PSE. On average, the level of precision in the two experimental conditions appears to be very similar. To assess the possibility of a different level of precision in the two experimental conditions, we performed an independent-sample t-test. The results of this test however showed no significant difference in precision between Exp. 1a and Exp. 1b (t(28) = 0.29, p = 0.77). This in turn shows that although the two experimental conditions differed in the way the interval duration is marked (i.e., by briefly-flashed textures in Exp. 1a, and by brief blinks of the dot-array itself in Exp. 1b), the participants were equally sensitive to duration irrespective of this difference.


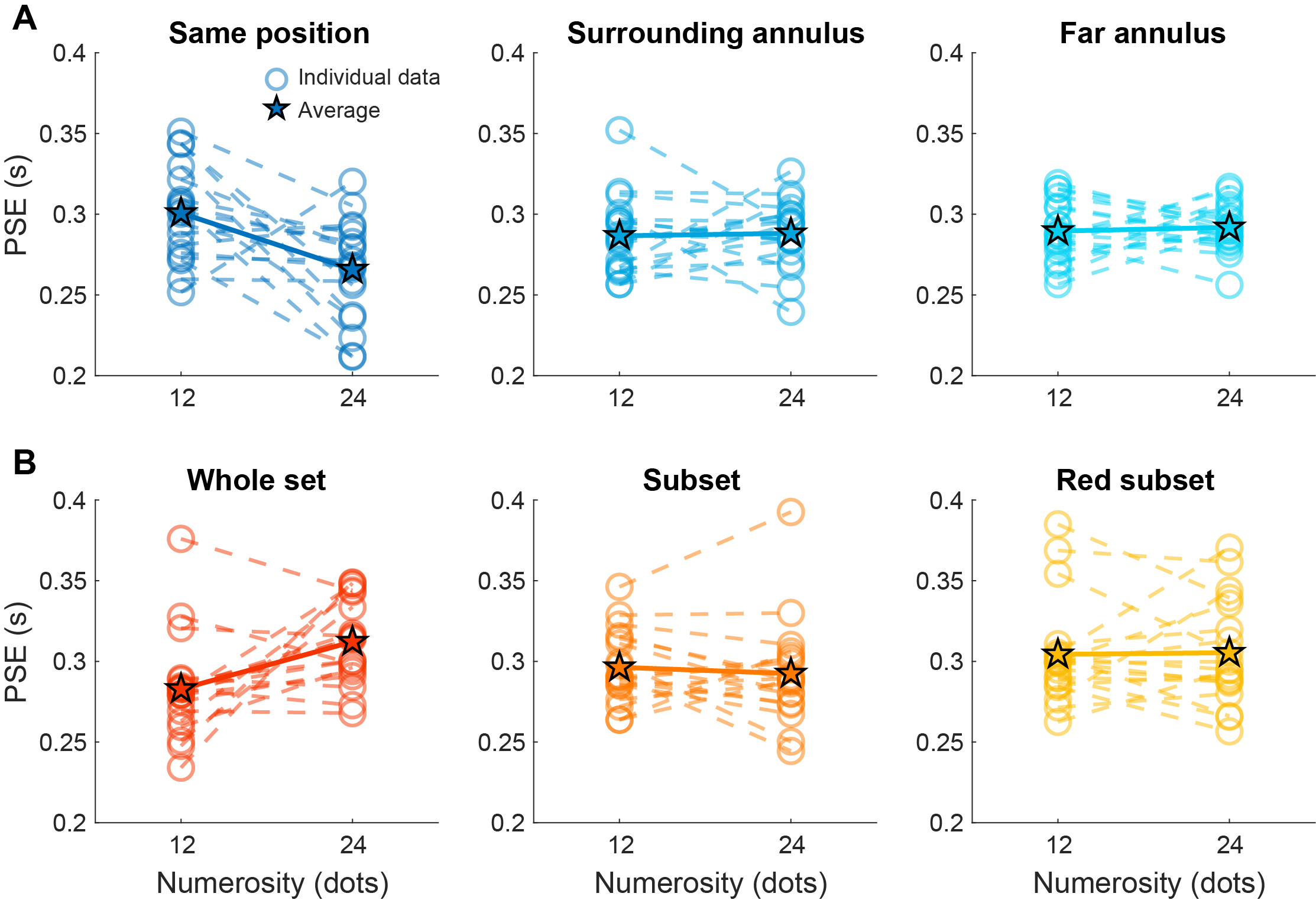


***Figure S3 – Individual PSEs in Exp. 2.*** *(A) PSEs in Exp. 2a, respectively for the “same position,” “surrounding annulus,” and “far annulus” condition (from left to right). Circles connected with dashed lines indicate the PSE values obtained from individual participants as a function of the reference numerosity. Stars indicate the average PSE as a function of numerosity. (B) PSEs in Exp. 2b, respectively for the “whole set,” “subset,” and “red subset” condition (from left to right).*


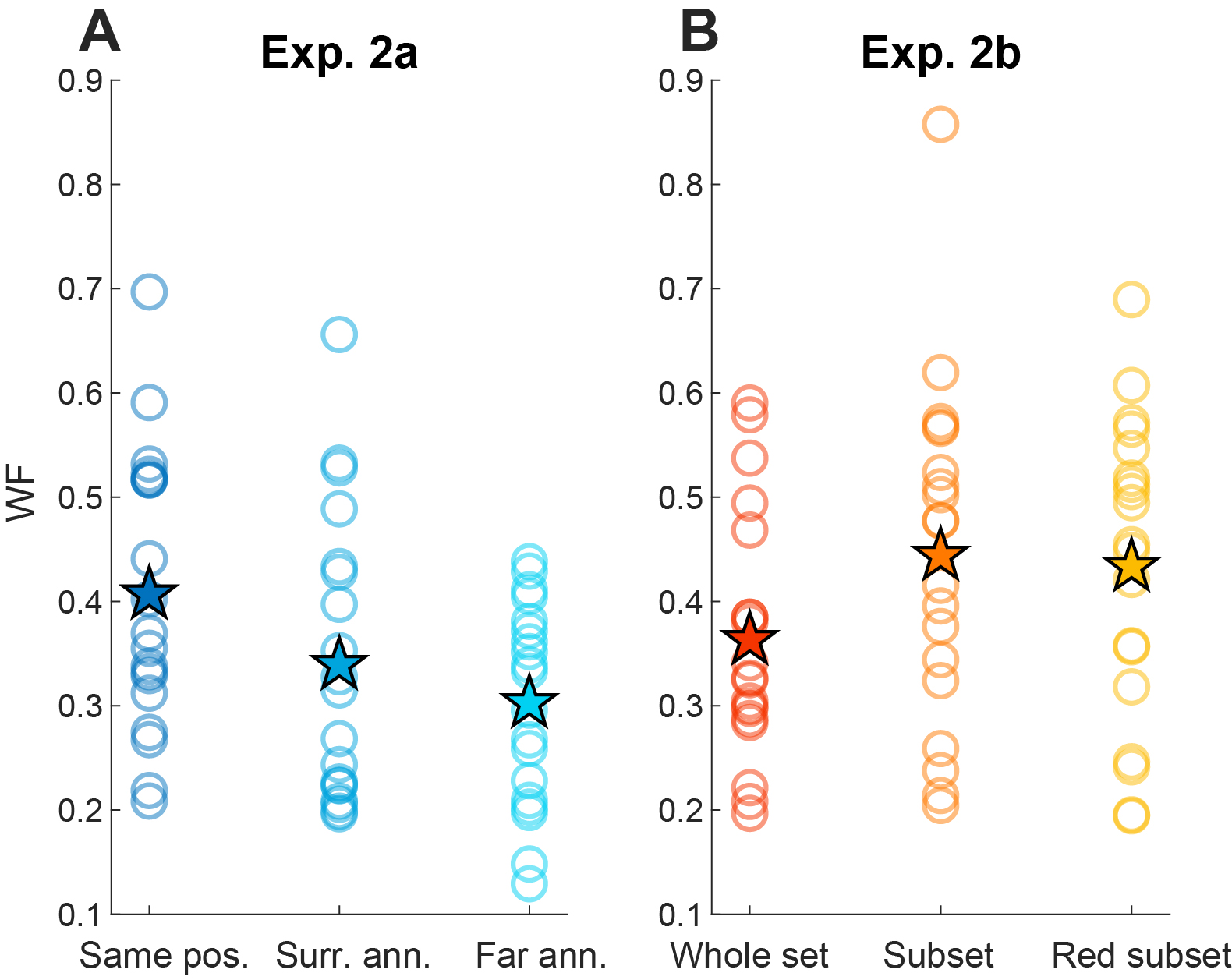


***Figure S4 – Weber’s fractions (WFs) in Exp. 2.*** *(A) WFs in Exp. 2a, as a function of the different stimulus conditions. Circles indicate individual measures of integration index, while stars indicate the average in each stimulus condition. (B) WFs in Exp. 2b.*

To assess the pattern of participants’ precision in the task across in Exp. 2a and 2b, we performed a one-way repeated measures ANOVA, with factor “stimulus condition” within each experiment. In Exp. 2a, the results of the ANOVA showed a significant main effect of stimulus condition (F(2,36) = 15.11, p < 0.001, η^2^_p_ = 0.45), suggesting that WF decreases (i.e., precision increases) when the dot-array is displaced away from the area of the interval markers. A series of paired t-tests further showed that WF in the “same position” condition are significantly higher than both the “surrounding annulus” (t(18) = 3.51, p = 0.002) and “far annulus” (t(18) = 5.42, p < 0.001) condition, but no significant difference was observed between these latter two conditions (t(18) = 1.90, p = 0.065). These results show that when there is an overlap between the dot-array and the interval markers, sensitivity to duration is lower compared to when the dot-array is displayed in a non-overlapping position. In Exp. 2b, we found again a significant main effect of stimulus condition (F(2,36) = 4.90, p = 0.013, η^2^_p_ = 0.21). The pattern is however opposite in this case, with WFs increasing (i.e., precision decreasing), when the intervals are marked by only a subset of the dots. A series of paired t-tests showed that WFs are significantly lower in the “whole set” condition compared to both the “subset” (t(18) = 2.88, p = 0.020) and “red subset” (t(18) = 2.50, p = 0.034) condition. No significant difference was observed between the “subset” and “red subset” condition (t(18) = 0.37, p = 0.71). These results show that having only a limited number of items blinking to mark the intervals limits the sensitivity to duration. Surprisingly though, making the subset more salient and clear even before the onset of the intervals does not seem to increase the performance.
